# Water regime history drives responses of soil Namib Desert microbial communities to wetting events

**DOI:** 10.1038/srep12263

**Published:** 2015-07-21

**Authors:** Aline Frossard, Jean-Baptiste Ramond, Mary Seely, Don A. Cowan

**Affiliations:** 1 Centre for Microbial Ecology and Genomics (CMEG), Genomic Research Institute, University of Pretoria, Pretoria, South Africa; 2 Gobabeb Research and Training Centre (GTRC), PO Box 953, Walvis Bay, Namibia; 3 Animal, Plant and Environmental Sciences, University of the Witwatersrand, Johannesburg, South Africa

## Abstract

Despite the dominance of microorganisms in arid soils, the structures and functional dynamics of microbial communities in hot deserts remain largely unresolved. The effects of wetting event frequency and intensity on Namib Desert microbial communities from two soils with different water-regime histories were tested over 36 days. A total of 168 soil microcosms received wetting events mimicking fog, light rain and heavy rainfall, with a parallel “dry condition” control. T-RFLP data showed that the different wetting events affected desert microbial community structures, but these effects were attenuated by the effects related to the long-term adaptation of both fungal and bacterial communities to soil origins (i.e. soil water regime histories). The intensity of the water pulses (i.e. the amount of water added) rather than the frequency of wetting events had greatest effect in shaping bacterial and fungal community structures. In contrast to microbial diversity, microbial activities (enzyme activities) showed very little response to the wetting events and were mainly driven by soil origin. This experiment clearly demonstrates the complexity of microbial community responses to wetting events in hyperarid hot desert soil ecosystems and underlines the dynamism of their indigenous microbial communities.

Arid soil systems constitute the most extensive and one of the harshest terrestrial biomes on Earth. The central Namib Desert (Southwest Africa) is thought to have exhibited hyperarid conditions for the last 5 million years, making it the oldest hyperarid desert on the planet^1^. Its surface temperature is highly variable (0–50 °C)^2^, and it receives an annual rainfall ranging between 5 and 18 mm^2^. Large differences in longitudinal water availability across the Namibian gravel plain (ca. 200 km) create an aridity gradient which has been shown to affect the assembly of soil and hypolithic bacterial communities^3^.

The central Namib Desert environment is characterized by two principal sources of water: fog and rainfall. Near the coast, fog provides an important source of available water, and penetrates inland as far as ca. 60 km^2^. Rainfall events are sporadic in the central Namib Desert, but recent models predict an increase in precipitation intensities (up to 25%) and longer dry periods between rain events^4^. However, in parallel, the variability of rain event intensities and timing is also expected to increase in this region^5^.

The scarcity of available water in hyperarid environments creates a range of niches occupied by specialized organisms capable of surviving low water activity conditions^6^,^7^,^8^. Water stress has been shown to strongly affect microbial metabolism^9^, which in term dictates ecosystem function^9^,^10^.

The frequency of daily and seasonal precipitation events has been shown to be closely related to microbial activity^11^. Extended dry periods can result in high cellular mortality, due to desiccation and oxidative damage^7^. However, when water becomes available, microbial activity may shift from water to resource limitation^11^. The magnitude of a water pulse event is often correlated to the ecological response it triggers^12^, particularly in terms of ecosystem net nutrient gain or loss^10^. While small rain events simply redistribute materials and reallocate resources in the soil^13^, larger magnitude rain events may lead to nutrient gains or losses by activating metabolic reactions of certain organisms^11^.

Drying-rewetting cycles (i.e. drought periods followed by wetting events) have been shown to influence the composition of microbial communities and to subsequently affect ecosystem functions^14^,^15^. In temperate-region soils, microbial communities experiencing regular episodic rainfalls were found to be more tolerant to water regime change than communities that did not experience regular rain events, such as those present in desert soils^15^,^16^,^17^. Furthermore, water regime history has been observed to affect both function and composition of microbial communities^15^,^16^,^18^,^19^.

Rainfall and fog events in desert systems are highly unpredictable, making it difficult to study their impacts *in situ*. The objective of this investigation was therefore to assess the response of bacterial and fungal microbial communities from Namib Desert soils with distinct water regime histories (i.e. ephemeral riverbed which are repeatedly flooded^2^,^20^,^21^ and gravel plains, never flooded) when pulsed with wetting events of different timings and intensities (mimicking fog, light rain and heavy rain) which could be related to potential/modeled climatic discrepancies linked to global changes. We hypothesized that edaphic microbial communities originating from environments with higher frequencies and intensities of dry-wet cycles (such as riverbeds) would be more resilient, in term of microbial diversity and functioning, than those from habitats with less environmental variation (such as gravel plain soil). We also hypothesized the timing more than the intensity of wetting events to be the main microbial community shift driver.

## Materials and Methods

### Experimental design

A full factorial design, resulting in a total of 168 microcosms (2 soil origins * 3 replicated sampling sites * 4 treatments * 7 sampling times) was implemented. The microcosms were filled with soils with different water regime histories (desert *Riverbed* or *Gravel plain* soils). Three distinct water treatments simulating different wetting events occurring naturally in the Namib Desert were used: 1) *Fog (F)*, 2) *Light Rain (LR)*, and 3) *Heavy Rain (HR)*. In addition, a dry *Control (C)* condition was also established. Each soil origins and treatment combination was replicated 3 times. The microcosms consisted of Perspex® cylinders (4 × 5.5 cm) with a base made of aluminum foil perforated with 14 small holes made with a sterile surgical needle (0.8 mm diameter).

**Figure 1:**
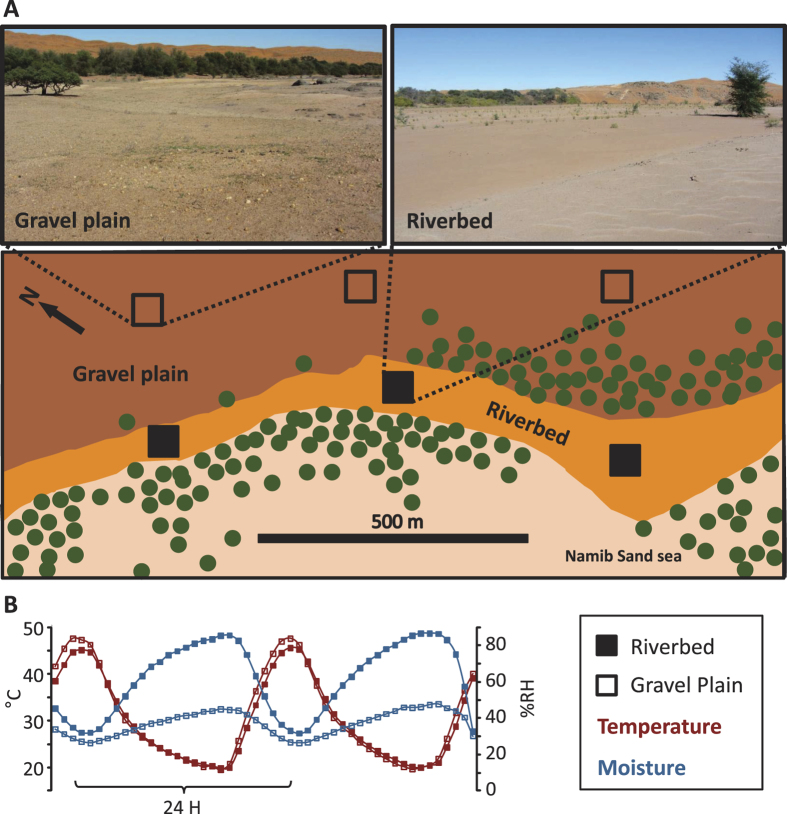
(**A**) Pictures and scheme representing the replicated sampling sites in the riverbed (n = 3, filled squares) and in the gravel plain (n = 3, empty squares). Green circles represent vegetation. (**B**) Averaged temperature and humidity variation of the riverbed and gravel plain soils over a 48h period (n = 3 for each soil origins). Photographs: Aline Frossard.

The microcosms were filled with shallow subsurface soils (2–10 cm deep) collected in the vicinity of the Gobabeb Research and Training Center in Namibia. Both riverbed and gravel plain soils were collected at 3 replicated sites, each ca. 500 m apart (Fig.1). The three riverbed soils were collected in the Kuiseb Riverbed (S23°33.726 E15°01.990; S23°33.395 E15°01.866; S23°33.231 E15°01.681) and the three gravel plain soil samples adjacent to the Kuiseb River (S23°33.520 E15°02.181; S23°33.308 E15°01.953; S23°33.139 E15°01.749). All soils were collected on the same day (April 24^th^, 2013), between 10 am and 12 am. Soil subsurface (2 cm deep) temperature and humidity were recorded prior to sampling at each sampling site every 10 min for 2 days using iButtons (Fairbridge Technologies, Wendyhood, South Africa; Fig. 1). Each soil was sieved to 2 mm, placed in sterile Whirl-Pak® sampling bags (Nasco, Wisconsin, USA) and subsequently stored in a dark low humidity environment at ambient temperature for 4 days prior to filling the microcosms. Microcosm vessels were washed, sterilized, filled with 50 g of riverbed or gravel plain soil and randomly placed on racks in constant temperature dark incubators at 28 °C (corresponding to the Gobabeb annual average daily-max temperature) for the entire period of the experiment (36 days). The microcosms were pre-incubated for 8 days to avoid artifacts in the analysis due to soil settlement and temperature equilibration.

**Figure 2:**
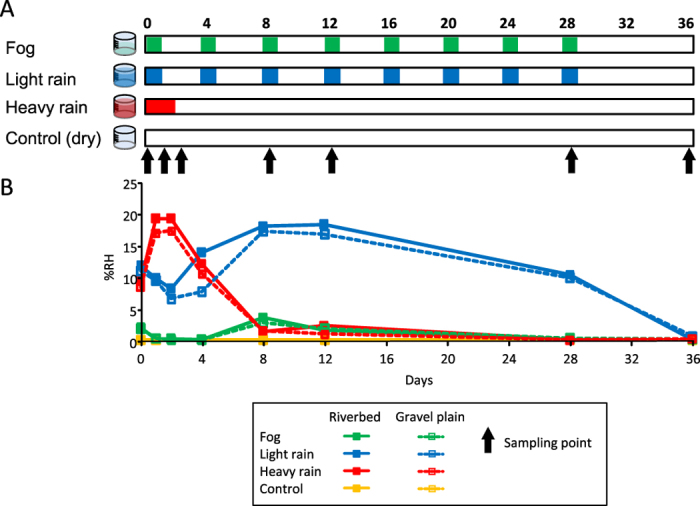
(**A**) Experimental setup representing the day when each wetting event treatment (*F, LR, HR* and *C*) was applied over the 36 days (sampling times are indicated with arrows) and (**B**) graph of relative humidity (%RH) in the microcosms.

An artificial “Namib desert precipitation solution” was synthesized, based on the averaged chemistry data of fog and rain occurring around the Gobabeb research station^2^ and consisting of: 2.5 ppm 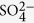 , 2 ppm 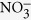, 3 ppm Cl^−^, 1.8 ppm Na^+^, 0.3 ppm K^+^, 1 ppm Ca^2+^, 0.4 ppm Mg^2+^, 0.4 ppm 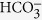, and 0.9 ppm 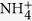. Three different water treatments with different intensities and frequencies observed in the Namib Desert (Fig. 2; rates based on Eckardt *et al.*^2^) were simulated: 1) *Fog (F)*: 15 mm (corresponding to 16 ml) rain-water month^−1^ delivered in 8 events (every 4 days); 2) *Light Rain (L)*: 60 mm (corresponding to 64 ml) rain-water month^−1^ delivered in 8 events (every 4 days); 3) *Heavy Rain (H)*: 60 mm (corresponding to 64 ml) rain-water month^−1^, concentrated in a single event (every 4 h over 36 hours). In parallel, a *Control (C)* with no “precipitation solution” addition was prepared. In order to compare the effect of frequency and intensity of water pulses among the treatments, the frequency of wetting events for *F* and *LR* treatments were similar throughout the experimental period. Similarly, the total quantity of water received between *LR* and *HR* treatments was similar at the end of the experimental period, although their wetting event frequencies differed (*LR* = 8 events, *HR* = 1 event).

### Incubation and Sampling

168 samples were recovered from the microcosms at 4 h, 1, 2, 8, 12, 28, and 36 days after the first wetting event was applied.) Samples were collected exactly 4 hours after the treatment application when a sampling date matched a wetting event (Fig. 2). At each sampling time, soil microcosms (one for each origin, treatment and replicate) were collected (destructive sampling) and mixed thoroughly. Subsamples were immediately stored at 4 °C for physico-chemical analyses, at −80 °C for metagenomic DNA extraction, and at −20 °C for enzyme assays. The residual soil was weighed, dried overnight at 105 °C and re-weighed to calculate the water content.

### Edaphic parameters

19 physico-chemical parameters were determined for each soil origins (×3 replicated sampling sites; Fig. 1A). The soil water retention capacity (WRC) was determined using pressure plate extractors and pressures of 150 and 1000 kPa. It was calculated using this equation ‘WRC = dθ/dh’, where ‘dθ’ represents the difference of water quantity retained by the soil between the pressures and ‘dh’ the difference of pressure applied in the extractors^22^. Soil pH was measured in soil slurries (1:2.5 soil/deionised water ratio) with a pH meter (Crison basic 20, Barcelona, Spain). Soil carbon content (%) was measured according to Nelson (1982)^23^. 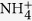 and 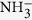 were extracted and quantified by titration and steam distillation according to Keeney and Nelson (1982)^24^. Extractable phosphorus was quantified using the Bray-1 method^25^. Cation exchange capacity (CEC) was determined according to Rhoades^26^. Salts (sodium, potassium, magnesium, calcium and sulfur), extracted according to Rhoades^26^, were analyzed using inductively coupled plasma atomic emission spectroscopy (ICP-AES; Spectro Genesis, Spectro Analytical Instruments GmbH, Germany). Soil texture was determined following the American Society for testing and Materials^27^ and the Bouyoucos^28^ protocols. Daily soil surface temperature range (DTR) and daily relative humidity range (DHR) values were calculated from iButton data as daily maxima minus daily minima.

### Enzyme Assays

Activities of six extracellular enzymes were assessed following the adapted protocol of Frossard *et al.*^29^ using chromogenic substrate analogues (p-nitrophenol: pNP): alkaline phosphatase (AP), leucine aminopeptidase (LAP), β-glucosidase (BG), β-N-acetylglucosaminidase (NAG), and phenol peroxidase (PP). Assays involved (i) placing 3 g of soil in 100 ml of autoclaved 5 mM Tris buffer (pH 8.3), (ii) stirring the slurry on a magnetic stirrer at constant speed, and (iii) adding 100 μl to 96-well microplates. Substrate analogues (4-nitrophenyl phosphate for AP, 4-Nitrophenyl-β-D-glucopyranoside for BG, 4-nitrophenyl-N-acetyl-β-D-glucosaminide for NAG, leucine p-nitroanilide for LAP, and L-3,4-dihydroxyphenylalanine for PO and PP) were added to the microplates at saturation level (100 μl of 5 mM for BG, NAG, and LAP assays; 100 μl of 10 mM for AP, PO and PP assays; based on previous tests, data not shown). Absorbance was measured after incubation at 28 °C for 3 h (NAG, LAP, PO, and PP) or 4 h (AP and BG) under constant agitation on a microplate reader (Mulitskan GO, Thermo Scientific, Waltham, USA) at 405 nm for AP, BG, NAG, and LAP assays and at 460 nm for PO and PP assays. Absorbance in soil sample controls (containing soil samples and buffer only) and substrate control (containing substrate only) was also measured in order to calculate a background absorbance, deducted from sample absorbance values in the calculation of catalytic rates.

In addition, fluorescin di-acetate degradation (FDA; used as a proxy of general bacterial metabolic activity) was determined following the protocol of Green *et al.* 2006^30^. Briefly, 0.5 g of soil was mixed with 12.5 ml of sterile PBS buffer, pH 7.4 and 0.25 ml of 4.9mM FDA dissolved in acetone, and incubated at 28 °C for 1 h under constant agitation. FDA hydrolysis was terminated by mixing 40 μl of acetone with 1 ml of soil slurry. After centrifugation (8800 × *g*, 5 min.) fluorescence was measured with a portable fluorometer (Quantifluor, Promega, Madison, USA).

### Bacterial and Fungal community analyses

Soil metagenomic DNA was extracted with the PowerSoil® DNA Isolation kit (MO BIO Laboratories, Carlsbad, CA, USA) following the manufacturer’s instructions, and stored at −20 °C. Bacterial 16S rRNA gene fragments were amplified using the fluorescent labeled forward-primer 341F-FAM: 5’6-FAM-CCTACGGGAGGCAGCAG-3’^31^ and reverse primer 908R: 5’- CCGTCAATTCMTTTGAGTTT-3’^32^. The PCR reaction mix (50 μl) contained (final concentration): 1× DreamTaq reaction buffer (Fermentas, USA), 0.2 mM dNTPs (Fermentas, USA), 0.5 μM of each primer, 0.1 mg ml^−1^ bovine serum albumin (BSA), 0.025 U μl^−1^ Dream Taq® (Fermentas, USA), and 1 μl of DNA extract. The initial DNA denaturation was at 95 °C for 5 min. Each of the following 25 amplification cycles involved a denaturation step at 95 °C for 30 sec, primer annealing at 55 °C for 30 sec, and an extension phase for 1.5 min at 72 °C. The final extension occurred at 72 °C for 10 min.

Fungal ITS regions were amplified using the fluorescent labeled forward-primer ITS1F-FAM: 5’-6-FAM-CTTGGTCATTTAGAGGAAGTAA-3’ and the reverse primer ITS4: 5’-TCCTCCGCTTATTGATATGC-3’^33^. PCR reaction mix (50 μl) contained (final concentration): 1× DreamTaq reaction buffer (Fermentas, USA), 0.2 mM dNTPs (Fermentas, USA), 0.4 μM of each primer, 0.1 mg ml^−1^ bovine serum albumin (BSA), 0.02 U μl^−1^ Dream Taq® (Fermentas, USA), and 2 μl of DNA extract. The initial DNA denaturation was at 95 °C for 5 min. Each of the following 28 amplification cycles involved a denaturation step at 95 °C for 1 min., primer annealing at 55 °C for 50 sec., and an extension phase for 1 min and 45 sec at 72 °C. The final extension was at 72 °C for 7 min.

PCR products were purified using the NucleoSpin® Gel and PCR Clean-up kit (Macherey-Nagel, GmbH & Co. KG, Düren, Germany) and the amount and quality of amplicons estimated spectrophotometrically (Nanodrop2000, Thermo Scientific, Waltham, USA). 200 ng of 16S rRNA gene amplicons were digested with the *HaeIII* restriction enzyme (Fermentas, USA) at 37 °C for 6 hours and 400 ng of ITS or 16S amplicons were digested with the Fastdigest *MspI* restriction enzyme (Fermentas, USA) at 37 °C for 1 hour. Digested amplicons were purified with the NucleoSpin® Gel and PCR Clean-up kit (Macherey-Nagel, GmbH & Co. KG, Düren, Germany) and 4 μl of digested DNA was mixed with 6.75 μl of formamide and 0.25 μl of GeneScan^TM^ 600 Liz® size standard v2.0 (Applied Biosystems, Foster City, USA) prior to denaturation for 5 min at 95 °C. Electrophoretic separation of terminal-restriction fragments (T-RFs) was conducted using an ABI3130XL sequencer (Applied Biosystems). True peaks and fragments of similar size were identified and binned using the softwares R^34^ and Perl^35^.

### Data Analysis

Response variables included extracellular enzyme activities and bacterial and fungal community diversity indices. Treatments (i.e. *F*, *LR*, *HR* and *C*), soil origins (i.e. *riverbed* and *gravel plain*) and sampling times (i.e. 4 h, 1, 2, 8, 12, 28 and 36 days) were treated as fixed factors. QQ-plots and frequency histograms indicated that residuals did not meet the assumptions required for parametric tests. Therefore, variables (x) were transformed according to ln (x + 1) to achieve normality. Linear models were used to determine significance values between soil origins for the different edaphic parameters using the function *lm* implemented in the software R^34^. Non-metric multidimensional scaling (NMDS) analyses were performed with the function *meta.mds* of the package *vegan* implemented in R. Permutational multivariate analysis of variance (PERMANOVA) with either soil origins, treatments or sampling date as factors was performed with the function *adonis* in R (vegan package). Calculations were based on Bray-Curtis distances and 1000 permutations. Planned contrasts reporting differences among treatments were assessed with the function *adonis* comparing selected combinations of the four water treatments: *C* vs. *F*, *C* vs. *LR*, *C* vs. *HR*, *F* vs. *LR*, *F* vs. *HR*, *LR* vs *HR*. Edaphic factors were fitted onto the ordinations by using the function *envfit* of the R package *vegan*. Significance of the associations was determined by 1000 random permutations. The Bray-Curtis distances between C and the different treatment (i.e. F, LR and HR) communities were used to calculate the resilience index ‘Res_t_’ using this equation ‘Res_t_ = (2 D_t_/(D_0_ + D_t_)) −1’^36^, where D_t_ = distance between C and each treatment at time ‘*t*’ and D_0_ = distance between C and each treatment after 4 h. This index varies between −1 and 1, where a value of 1 means full recovery (maximal resilience) whereas a value of 0 indicates no recovery and negative values imply that D_t_ is higher than D_0_, indicating a shift of the treated community in opposite direction to the control community at time ‘t’.

## Results and Discussion

### Soil physico-chemical parameters

This microcosms experiment was designed to assess the effect of different water regimes on desert soil microbial communities by mimicking different wetting events naturally occurring in the Namib Desert^2^. We evaluated the responses for soils of two different origins; i.e. riverbed and gravel plain soils (Fig. 1), which have known different water regime histories^2^,^21^. The daily relative humidity range (DHR) was more than 2 fold higher in the riverbed soils than in the gravel plain soils (Fig. 1, Table 1) reflecting the persistent presence of shallow groundwater in the former^20^.

Long-term flooding data from the Gobabeb Research and Training Centre (GRTC; within 2 km from the sampling sites) support the conclusion that the two soils exhibit substantially different water regime histories. In the past 15 years, the riverbed in the vicinity of Gobabeb has flooded up to 190 days per annum, with an average of 25 flooding days per annum (data from the GRTC). Moreover, the first paleoflood evidences date from the 13^th^ century^21^. A significant volume of groundwater has been shown to flow at shallow depth (between 0 and 5.5 m, depending on the intensity of flooding events) in the alluvial aquifer of the Kuiseb riverbed^20^. In comparison, only low volume of groundwater flows along the 1% gradient of the Namib gravel plains, soils after rain events, where elevated evaporation rates prevail^2^.

The two soil origins also differed significantly in their *in situ* daily temperature ranges (DTR), where the mean daily temperature was significantly higher in the shallow subsurface gravel plain soils than in the riverbed soils. The soil texture contrasted between the two soils, with the riverbed soil containing significantly less coarse sand than the gravel plain soil (Table 1). Soil grain-size distribution in ephemeral riverbed channels is known to be redistributed at each flood event^37^, which can have a substantial effect both on the composition and activity of microbial communities in arid systems^38^,^39^. As a consequence, water retention capacity was found more than two times higher in the riverbed than in the gravel plain (Table 1). Gravel plain and riverbed soils further diverged in their total phosphorus content, which was significantly higher in the gravel plain soils (Table 1). Phosphorus is a limiting factor for microbial growth in soils, and variations in available phosphorus in arid soils have been shown to modify soil microbial functional and structural properties^40^. Moreover, phosphorus availability was also shown to be impacted by soil moisture in weathered soils^41^ and by the intensity of desert flashfloods in arid ecosystems^42^. Therefore, all physico-chemical factors segregating the two soils could directly be linked to soil moisture variation. This thus supports the fact that water regime history has led to differences between the riverbed and gravel plain soil physico-chemistries. However, the effect of water regime history might be balanced by several salts, such as Ca^+^, K^+^, Na^+^ as well as S content, which all presented substantial variations between the riverbed and gravel plain soils, although not significantly different (p > 0.05). As an extreme example, it has indeed been shown that even minor differences in sand crystal composition can lead to completely distinct microbial community structures^43^.

### Microbial Activities

The activities of various extracellular enzymes showed marked differences between the two soil origins (Table 2). Over the entire length of the experiment, BG and LAP activities were significantly higher in the gravel plain samples than in the riverbed samples, while NAG activity was greater in the gravel plain at all sampling dates except the last (Table 2). Only AP and NAG activities were significantly affected by the water treatments at day 12 for AP and NAG (Table 2).

These results show that extracellular enzyme activities are retained in Namib Desert soils even when soils are in a dessicated state (i.e. dry control, Fig. 2B). Soil enzymes have been previously shown to be active at very low water contents^44^,^45^, and may play a role in maintaining long-term nutrient availability in soils. Consequently, rather than being limited by metabolic constraints of microorganisms, it is thought that only diffusion limitations may control enzyme activities in dry soils^44^,^45^.

### Impact of water regime histories on fungal and bacterial community structure

Although the method used (t-RFLP) did not allow distinguishing for relative abundance in fungal or bacterial species composition, it was precise enough to detect shifts in the fungal and bacterial community structures. When all samples (all treatments, soil origins, and sampling times) were combined in a same PERMANOVA analysis, statistically significant differences between to the two soil origins were observed in the fungal community structure over the entire length of the experiment (p < 0.05 for all sampling times combined and for each sampling time individually, Table 3). In the bacterial community structure, significant differences between the riverbed and the gravel plains were also found at all sampling dates but day 12 (Table 3). This clearly indicates that the edaphic environment, and in particular the water regime history of gravel plain and riverbed soils, was a critical factor driving both fungal and bacterial community structures. All 18 soil physicochemical parameters measured were significantly linked to the fungal community NMDS ordination, and all but Corg, Mg, Na and S significantly fitted the bacterial community ordination (supp. Table 1).

**Figure 3:**
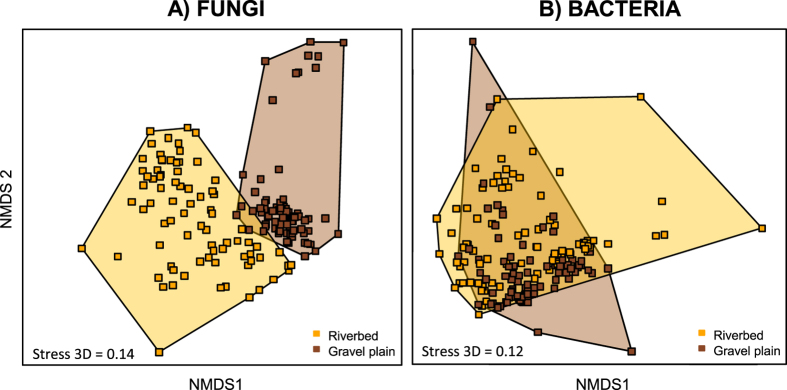
NMDS ordination of fungal and bacterial community structure inferred from OTUs relative abundance (obtained from T-RLFPs from all water treatments and all sampling times combined). Different clusters denotes for communities originated from the riverbed (orange) and the gravel plain (brown).

The divergence between the two soil origins was clearer in the fungal community where the riverbed and the gravel plain clusters were clearly separated and barely overlapped compared to the bacterial community where the two clusters largely overlapped (Fig. 3). The stronger association of the fungal community structure with the soil origin suggests that desert fungal communities were either more soil-specific than bacterial communities^46^ or were more resistant to wetting events^47^.

### Impact of wetting event treatments on fungal community structure

**Figure 4:**
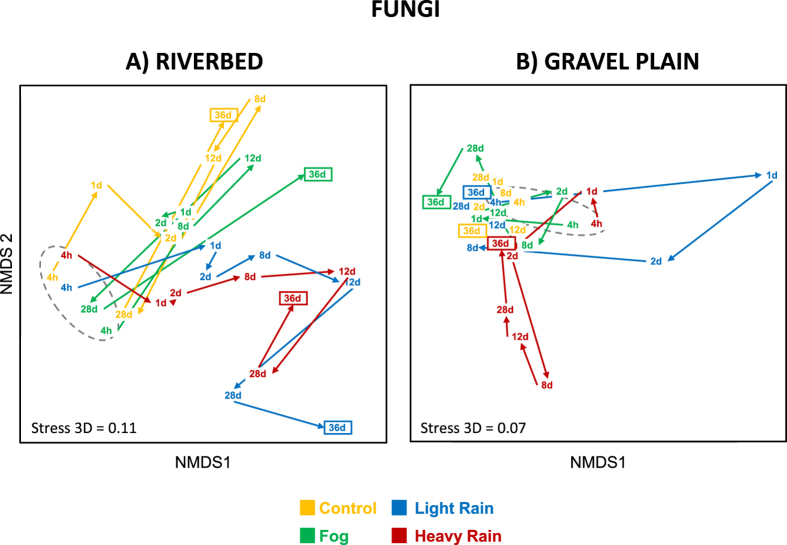
NMDS ordination of fungal community structure inferred from OTUs relative abundance (obtained from T-RLFPs) originated from the riverbed (**A**) and gravel plain (**B**) Different colors denotes for the different water treatments. Points in the ordination are mean of the 3 replicated microcosms per treatment and per sampling time (4 h, 1, 2, 8, 12, 28 and 36 days). Arrows between points revealed the shifting direction of each community between sampling time and grey dashed line surrounds the first sampling time of each treatment.

In the riverbed samples, fungal communities were significantly affected by the different water regimes only at the end of the experiment (i.e., 36 days; Fig. 4A; Supp. Table 2), when soil moisture content had drastically decreased in all the microcosms (Fig. 2). At day 36, the *LR* fungal communities differed significantly from the *C* and *F* communities (contrast C/LR: P = 0.04; contrast F/LR: P = 0.022; supp. Table 2; Fig. 4A). Contrastingly, the HR fungal community structure did not differ from the ones of the other water-impacted fungal communities. This result suggests that fungal communities are altered when a substantial amount of water is dispensed at multiple occasions, but not when delivered in a single event, such as a storm.

In the gravel plain, fungal communities were less affected by the water treatments (supp. Table 2, no significant different among treatment for all the sampling dates together). However, fungal communities were altered by *LR* treatment only 1 day after the start of the experiment (Fig. 4B; contrast C/LR: P = 0.022 and contrast F/LR: P = 0.024; supp. Table 2) and displayed a rapid reversion back towards their original structure (Fig. 4B), which suggest high levels of both responsiveness and resilience of these communities.

Microbial communities are generally sensitive to environmental disturbance^48^, with only a minority of studies reporting a recovery of the soil microbial community composition after a disturbance event (reviewed in Shade *et al.*^49^). Fungal community resilience towards the wetting events was only observed in the gravel plain soil (suppl. Fig. 1), where moisture level variations (i.e. DHR) were lower compared to the riverbed soils (Fig. 2B). This is in line with previously published observations, showing that a soil with more constant physico-chemical properties leads to a greater resilience capacity of the microbial communities^50^. Furthermore, this suggests that desert soil properties, such as water regime histories might have a direct effect on soil fungal community stability and ultimately on soil ecosystem functioning after disturbance^51^.

### Impact of wetting event treatments on bacterial community structure

**Figure 5:**
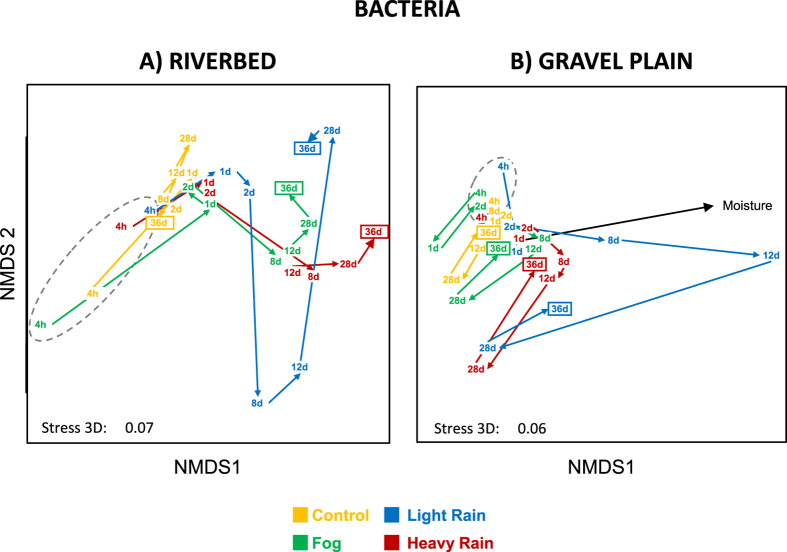
NMDS ordination of bacterial community structure inferred from OTUs relative abundance (obtained from T-RLFPs) originated from the riverbed (**A**) and gravel plain (**B**) Different colors denotes for the different water treatments. Points in the ordination are mean of the 3 replicated microcosms per treatment and per sampling time (4 h, 1, 2, 8, 12, 28 and 36 days). Arrows between points revealed the shifting direction of each community between sampling time and grey dashed line surrounds the first sampling date of each treatment. Black arrows indicates the direction of the experimental variable “moisture levels in the microcosms” significantly fitted with the bacterial community structure (permanova, P < 0.05).

The riverbed bacterial community structures in the *LR* microcosms differed significantly from the *C* communities from day 12 onwards (Fig. 5A; contrast C/LR: P = 0.018, 0.027, 0.015 at day 12, 28 and 36, respectively; supp. Table 3). Differences between *HR* and *C* community structures were observed from day 28 (Fig. 5A; contrast C/HR: P = 0.035 and 0011 at day 28 and 36, respectively; supp. Table 3).

In the gravel plain samples, bacterial community structures were only affected by the *LR* treatment (Fig. 5B). *LR* communities differed from the *C* communities at day 8, 12 and 36 (contrast C/LR: P = 0.001, 0.001 and 0.021 for day 8, 12 and 36 respectively) and from the *F* and *HR* communities at day 12 only (contrast F/LR: P = 0.022, contrast LR/HR: P = 0.005, Fig. 5B, supp. Table 3). The major shift of the bacterial community structure in the *LR* microcosms at day 12 was strongly correlated to soil moisture content (Fig. 5B; r^2^ = 0.316, P = 0.009).

Shifts in the bacterial community structure were detected only after the moisture in the microcosms had reached a high level, such as at day 12 in the *LR* treatment (Fig. 2B). This suggests that wetting events must exceed a certain intensity in order to overcome the effect of water regime histories and to induce structural changes in desert soil bacterial communities. This suggestion is consistent with published field studies, which demonstrated that adaptation of the microbial community structures to long-term soil environmental conditions^52^ might attenuate the effects of short-term environmental variation^15^.

## Conclusions

Altogether, this study demonstrates the responsiveness of shallow subsurface soil desert bacterial and fungal communities to water availability changes. Long-term environmental adaptation of both fungal and bacterial communities to soil origins with different soil water regime history were found to attenuate short-term responses towards disturbance caused by wetting events. However, when microbial communities responded to the wetting events, intensity (i.e. the total amount of water) rather than frequency of wetting events had the greatest impact on both bacterial and fungal community structures. This result contrasts with previous findings showing that precipitation frequency more than intensity influences biological crust growth and community succession^53^ and metabolic activities^54^ in arid region soils. However, successive wetting and drying cycles have also been shown to have a substantial effect on microbial community composition (e.g.^15^,^44^,^55^), with drought history strongly influencing active community composition during the next moisture pulse^56^. Thus, the intensity of each successive wetting event is expected to affect the microbial community structure increasingly after each drying and rewetting cycles.

According to recent climate change models, precipitation intensities, as well as the duration of dry periods between the wetting events, are projected to increase in southwest Africa^4^. These models are supported by some recovered data of intensification of extreme rainfall events in dryland ecosystems^57^. Rain-use efficiency (i.e. the ratio of annual net primary production to annual precipitation) in xeric systems has also been projected to increase in the future. The latter, together with increased rainfall intensity, may drive deeper percolation of soil water and will reduce soil evaporation, reducing loss of available water for biotic activity^5^. We thus suggest that if such projected precipitation trends are accurate, substantial changes in the structures of microbial communities in hot desert subsurface soils could be expected. However, while microbial community structures might be irreversibly altered by the successive dry and wet cycles, microbial activities are expected to be more resilient, suggesting functional redundancy of the microbial communities^48^. This notwithstanding, the findings of this study suggest that microbial community responses towards water regime change are complex, as suggested for other less arid soil systems^55^,^58^.

## Additional Information

**How to cite this article**: Frossard, A. *et al.* Water regime history drives responses of soil Namib Desert microbial communities to wetting events. *Sci. Rep.* **5**, 12263; doi: 10.1038/srep12263 (2015).

## Supplementary Material

Supplementary Information

## Figures and Tables

**Table 1:** Mean and standard errors of soil physico-chemical parameters for Riverbed and Gravel plain (N = 3).

	Soil origins		differences between soil origins	
	Riverbed	Gravel Plain	F	P
DTR (°C)	25.8 ± 0.6	28.4 ± 0.3	15.52	0.017*
DHR (%RH)	56.2 ± 3.5	22 ± 5.5	25.54	0.015*
WRC (g/kPa)	4.3 × 10^−5^ ± 5.5 × 10^−6^	1.6 × 10^−5^ ± 4.8 × 10^−8^	14.709	0.031*
pH	8.8 ± 0.3	8 ± 0.4	2.53	0.188
Organic C (%)	0.1 ± 0.0	0.1 ± 0.0	3.37	0.140
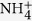 (μg g^−1^)	7.7 ± 1.0	8.3 ± 2.1	0.07	0.801
NO_3_^−^ (μg g^−1^)	6.6 ± 2.3	13.8 ± 4.9	1.74	0.257
P (μg g^−1^)	4.4 ± 0.4	9 ± 1.1	16.23	0.016*
CEC (cmol^+^ kg^−1^)	3 ± 0.2	3.5 ± 0.4	1.69	0.263
Ca^+^ (μg g^−1^)	724 ± 45.2	2235.7 ± 543.3	7.69	0.051
K^+^ (μg g^−1^)	101.1 ± 12.2	311.1 ± 97.5	4.57	0.099
Mg^+^ (μg g^−1^)	49.9 ± 5.9	69.7 ± 7.9	4.07	0.114
Na^+^ (μg g^−1^)	112 ± 71.5	381.5 ± 211.1	1.46	0.293
S (μg g^−1^)	19.5 ± 13.2	110.5 ± 86.4	1.08	0.356
Coarse sand (<2 mm; %)	0.10 ± 0.07	4.79 ± 0.56	70.06	0.001*
Medium sand (<630 μm; %)	49.8 ± 5.1	39.4 ± 10.4	0.81	0.417
Fine sand (<200 μm; %)	50.1 ± 5.1	55.8 ± 10.7	0.23	0.655
Silt (<50 μm; %)	0.002 ± 0.001	0.020 ± 0.009	3.74	0.125
Clay (<2 μm; %)	0.001 ± 0.000	0.015 ± 0.006	3.74	0.125

Mean ± standard deviation. DTR = Daily Temperature Range, DHR = Daily Humidity Range, WRC = Water retention capacity, F = F value, P = P value, Degree of freedom = 1, 5 (numerator, total).

**Table 2:** ANOVA table showing differences in extracellular enzyme activities between soil origins (i.e. riverbed and gravel plain), among treatments (i.e. F, LR, HR and C) and interactions between soil origins and treatments.

		BG		NAG		AP		LAP		PP		FDA	
	DF	F	P	F	P	F	P	F	P	F	P	F	P
**All sampling times**													
soil origins	1, 112	57.53	<0.001*	40.57	<0.001*	21.24	<0.001*	53.98	<0.001*	1.07	0.304	1.83	0.179
treatment	3, 112	0.95	0.412	0.56	0.643	0.74	0.528	0.23	0.876	1.31	0.274	1.48	0.225
sampling	6, 112	1.03	0.410	0.75	0.608	0.73	0.628	0.93	0.476	0.83	0.550	151.03	<0.001^*^
soil origins : treatment	3, 112	0.61	0.610	0.32	0.814	0.48	0.695	0.33	0.800	0.65	0.582	0.46	0.71
soil origins: sampling	6, 112	0.84	0.545	0.57	0.750	1.58	0.159	0.63	0.706	0.59	0.736	2.00	0.072
treatment: sampling	18, 112	0.35	0.993	0.65	0.848	0.86	0.621	0.70	0.807	0.90	0.580	0.39	0.987
soil origins: treatment: sampling	18, 112	0.36	0.992	0.83	0.667	1.036	0.427	0.83	0.660	0.79	0.707	0.56	0.921
**4h**													
soil origins	1, 16	7.31	0.016*	8.08	0.012*	7.11	0.017*	7.05	0.017*	0.41	0.533	0.62	0.440
treatments	3, 16	0.08	0.967	0.13	0.943	0.28	0.836	0.31	0.820	1.35	0.293	2.83	0.071
soil origins : treatments	3, 16	0.19	0.900	0.30	0.822	0.34	0.800	0.43	0.732	1.23	0.330	1.48	0.259
**1 day**													
soil origins	1, 16	15.67	0.001*	7.73	0.013*	10.08	0.006*	8.49	0.010*	0.00	0.995	1.75	0.205
treatments	3, 16	0.19	0.900	0.05	0.984	1.05	0.398	1.45	0.266	1.73	0.201	0.53	0.667
soil origins : treatments	3, 16	0.18	0.911	0.27	0.844	0.91	0.456	1.41	0.276	0.66	0.588	1.01	0.414
**2 days**													
soil origins	1, 16	16.89	<0.001*	5.26	0.036*	0.38	0.546	13.74	0.002*	0.00	0.964	0.00	0.964
treatments	3, 16	1.70	0.207	0.34	0.796	2.01	0.153	0.36	0.783	0.83	0.496	2.29	0.113
soil origins : treatments	3, 16	0.91	0.456	0.28	0.839	2.11	0.139	0.40	0.758	0.14	0.934	0.80	0.509
**8 days**													
soil origins	1, 16	5.54	0.032*	4.38	0.052	0.04	0.852	6.49	0.021*	2.34	0.146	4.63	0.047*
treatments	3, 16	1.07	0.391	0.90	0.464	0.82	0.501	1.02	0.409	0.19	0.903	0.00	1.000
soil origins : treatments	3, 16	0.60	0.627	0.93	0.447	0.35	0.787	0.69	0.569	3.60	0.037*	0.23	0.876
**12 days**													
soil origins	1, 16	24.48	<0.001*	20.35	<0.001*	35.01	<0.001*	6.72	0.020*	1.12	0.306	0.31	0.582
treatments	3, 16	2.11	0.139	4.88	0.013*	5.99	0.006*	0.75	0.539	0.54	0.663	0.43	0.732
soil origins : treatments	3, 16	2.33	0.112	4.79	0.014*	6.29	0.005*	1.23	0.329	0.06	0.978	0.95	0.438
**28 days**													
soil origins	1, 16	5.38	0.034*	5.46	0.033*	4.43	0.051	4.91	0.041*	0.63	0.434	3.66	0.074
treatments	3, 16	0.20	0.898	0.22	0.884	0.72	0.553	0.13	0.943	0.14	0.934	0.22	0.883
soil origins : treatments	3, 16	0.29	0.831	0.72	0.556	1.10	0.377	0.71	0.556	0.30	0.826	0.21	0.890
**36 days**													
soil origins	1, 16	12.69	0.003*	1.79	0.199	0.97	0.340	7.22	0.016*	0.24	0.630	0.57	0.462
treatments	3, 16	0.52	0.674	0.45	0.714	0.51	0.682	0.33	0.801	2.30	0.117	0.64	0.600
soil origins : treatments	3, 16	0.51	0.680	0.70	0.565	1.36	0.289	0.43	0.737	0.70	0.563	0.39	0.761

BG = β-glucosidase, NAG = β-N-acetylglucosaminidase, AP = Alkaline Phosphatase, LAP = Leucine aminopeptidase, PP = phenol peroxidase, FDA = Fluorescine di-acetate. DF = degree of freedom: numerator, total. F = F value. P = P value.

**Table 3:** PERMANOVA table showing differences in the bacterial and fungal community structures between soil origins (i.e. riverbed and gravel plain), treatments (i.e. F, LR, HR and C) and sampling times (4h, 1, 2, 8, 12, 28, and 36 days), and differences among soil origins and treatments at the different sampling times (all treatments combined).

	Fungal community			Bacterial community		
	DF	F	P	DF	F	P
**All sampling times**						
soil origins	1, 163	32.15	0.001*	1, 167	19.63	0.001*
treatment	3, 163	1.49	0.051*	3, 167	5.19	0.001*
sampling	6, 163	1.88	0.001*	6, 167	6.94	0.001*
soil origins : treatment	3, 163	1.43	0.057*	3, 167	1.68	0.038*
soil origins: sampling	6, 163	1.58	0.004*	6, 167	1.61	0.016*
treatment: sampling	18, 163	0.91	0.807*	18, 167	1.42	0.005*
soil origins: treatment: sampling	18, 163	0.88	0.905*	18, 167	0.72	**0.985**
**4 h**						
soil origins	1, 22	6.89	0.001*	1, 23	5.92	0.002*
treatments	3, 22	0.84	**0.666**	3, 23	0.50	**0.941**
soil origins: treatments	3, 22	0.95	**0.535**	3, 23	0.71	**0.755**
**1 day**						
soil origins	1, 23	8.98	0.001*	1, 23	3.14	0.021*
treatments	1, 23	1.63	**0.06**	3, 23	0.80	**0.666**
soil origins: treatments	1, 23	1.46	**0.115**	3, 23	0.51	**0.951**
**2 days**						
soil origins	1, 23	4.92	0.001*	1, 23	3.85	0.012*
treatments	1, 23	0.70	**0.923**	3, 23	0.63	**0.838**
soil origins: treatments	1, 23	0.71	**0.898**	3, 23	0.60	**0.839**
**8 days**						
soil origins	1, 23	2.87	0.001*	1, 23	4.13	0.003*
treatments	1, 23	0.83	**0.803**	3, 23	2.20	0.018*
soil origins: treatments	1, 23	0.84	**0.810**	3, 23	0.79	**0.663**
**12 days**						
soil origins	1, 23	4.63	0.001*	1, 23	1.79	**0.146**
treatments	1, 23	1.13	**0.294**	3, 23	4.60	0.001*
soil origins: treatments	1, 23	0.77	**0.859**	3, 23	1.29	**0.237**
**28 days**						
soil origins	1, 23	9.05	0.001*	1, 23	4.35	0.004*
treatments	1, 23	0.87	**0.616**	3, 23	2.42	0.003*
soil origins: treatments	1, 23	0.81	**0.676**	3, 23	1.05	**0.383**
**36 days**						
soil origins	1, 23	5.10	0.001*	1, 23	5.27	0.002*
treatments	1, 23	1.17	**0.214**	3, 23	2.73	0.004*
soil origins: treatments	1, 23	1.28	**0.130**	3, 23	1.01	**0.442**

F = F value, P = P value, DF = degree of freedom: numerator, total.
