# Supplementary material for: Water regime history drives responses of soil Namib Desert microbial communities to wetting events
Source: Sci Rep. 2015 Jul 21;5:12263. doi: 10.1038/srep12263 (PMC4508562; doi:10.1038/srep12263)
Supplement: Supplementary Information [file srep12263-s1.pdf]

Supplementary Information

Water regime history drives responses of soil Namib Desert microbial communities to wetting events

Aline Frossard[1], Jean-Baptiste Ramond[1], Mary Seely[2,3] and Don A. Cowan[1]

(1) Centre for Microbial Ecology and Genomics (CMEG), Genomic Research Institute, University of Pretoria, Pretoria, South Africa.

(2) Gobabeb Research and Training Centre (GTRC), PO Box 953, Walvis Bay, Namibia.

(3) Animal, Plant and Environmental Sciences, University of the Witwatersrand, Johannesburg, South Africa.

Supp. Table 1. Fit of soil physico-chemical parameters to the fungal and bacterial community structures. Fit determined by PERMANOVA. DTR = Daily Temperature Range, DHR = Daily Humidity Range, WRC = Water retention capacity.

	Fungi		Bacteria	
	r^2	P	r^2	**P**
DTR (°C)	0.50	**0.001***	0.29	**0.001***
DHR (%RH)	0.47	**0.001***	0.08	**0.003***
WRC (g kPa^{-1})	0.45	**0.001***	0.08	**0.006***
pH	0.43	**0.001***	0.09	**0.002***
Organic C (%)	0.36	**0.001***	0.34	0.100
NH_4^+ (µg g^{-1})	0.09	**0.002***	0.17	**0.001***
NO_3^- (µg g^{-1})	0.41	**0.001***	0.08	**0.003***
P (µg g^{-1})	0.64	**0.001***	0.13	**0.001***
CEC ($cmol^+$ kg^{-1})	0.31	**0.001***	0.08	**0.002***
Ca^+ (µg g^{-1})	0.46	**0.001***	0.07	**0.007***
K^+ (µg g^{-1})	0.52	**0.001***	0.07	**0.006***
Mg^+ (µg g^{-1})	0.45	**0.001***	0.01	0.386
Na^+ (µg g^{-1})	0.40	**0.001***	0.01	0.667
S (µg g^{-1})	0.32	**0.001***	0.00	0.816
Coarse sand (<2 mm; %)	0.63	**0.001***	0.13	**0.001***
Medium sand (<630 µm; %)	0.32	**0.001***	0.21	**0.001***
Fine sand (<200 µm; %)	0.17	**0.001***	0.21	**0.001***
Silt (<50 µm; %)	0.54	**0.001***	0.12	**0.001***
Clay (<2 µm; %)	0.42	**0.001***	0.07	**0.007***

Supp. Table 2. PERMANOVA table showing differences in the **fungal** community structure among treatments and specific contrasts of treatments for all sampling dates and at each sampling times separately for riverbed and gravel samples. DF = degree of freedom: numerator, total. F = F value. P = P value.

	**Factor / Contrast**	**Riverbed**			**Gravel Plain**		
		DF	F	P	DF	F	P
All dates	Treatment	3, 79	1.381	**0.049***	3, 83	1.518	0.079
	Sampling	6, 79	1.905	**0.001***	6, 83	1.415	0.054
	Treatment : sampling	18, 79	0.734	1.000	18, 83	1.143	0.198
4 h	Treatment	3, 10	0.592	0.940	3, 11	1.304	0.309
	C vs. F	1, 10	0.819	0.577	1, 11	2.471	0.074
	C vs. LR	1, 10	0.452	0.890	1, 11	0.452	0.831
	C vs. HR	1, 10	0.263	0.990	1, 11	0.547	0.760
	F vs. LR	1, 10	1.802	0.125	1, 11	2.813	**0.022***
	F vs. HR	1, 10	1.004	0.407	1, 11	0.957	0.363
	LR vs. HR	1, 10	0.550	0.833	1, 11	0.603	0.701
1 day	Treatment	3, 11	0.695	0.843	3, 11	2.989	0.068
	C vs. F	1, 11	0.785	0.595	1, 11	0.118	0.954
	C vs. LR	1, 11	1.143	0.298	1, 11	6.078	**0.022***
	C vs. HR	1, 11	0.635	0.800	1, 11	0.778	0.446
	F vs. LR	1, 11	0.415	0.959	1, 11	5.909	**0.024***
	F vs. HR	1, 11	0.768	0.621	1, 11	0.786	0.415
	LR vs. HR	1, 11	0.722	0.704	1, 11	1.777	0.192
2 days	Treatment	3, 11	0.652	0.969	3, 11	0.767	0.753
	C vs. F	1, 11	0.571	0.937	1, 11	0.773	0.611
	C vs. LR	1, 11	0.557	0.924	1, 11	1.129	0.294
	C vs. HR	1, 11	0.855	0.616	1, 11	0.251	0.971
	F vs. LR	1, 11	0.616	0.879	1, 11	0.865	0.569
	F vs. HR	1, 11	0.567	0.930	1, 11	0.805	0.598
	LR vs. HR	1, 11	1.060	0.366	1, 11	1.048	0.342
8 days	Treatment	3, 10	0.757	0.877	3, 11	0.935	0.584
	C vs. F	1, 10	0.965	0.505	1, 11	0.797	0.693
	C vs. LR	1, 10	0.913	0.545	1, 11	0.534	0.954
	C vs. HR	1, 10	0.990	0.443	1, 11	1.293	0.210
	F vs. LR	1, 10	0.843	0.607	1, 11	0.602	0.904
	F vs. HR	1, 10	0.670	0.848	1, 11	1.328	0.156
	LR vs. HR	1, 10	0.486	0.972	1, 11	1.186	0.288
12 days	Treatment	3, 9	0.881	0.676	3, 11	0.964	0.525
	C vs. F	1, 9	0.969	0.490	1, 11	0.387	0.950
	C vs. LR	1, 9	1.135	0.284	1, 11	0.690	0.656
	C vs. HR	1, 9	0.639	0.901	1, 11	1.522	0.147
	F vs. LR	1, 9	0.950	0.481	1, 11	0.617	0.734
	F vs. HR	1, 9	0.787	0.751	1, 11	1.501	0.183
	LR vs. HR	1, 9	1.064	0.358	1, 11	1.215	0.284
28 days	Treatment	3, 11	0.941	0.571	3, 11	0.693	0.872
	C vs. F	1, 11	0.253	0.998	1, 11	0.478	0.922
	C vs. LR	1, 11	1.153	0.319	1, 11	0.383	0.961
	C vs. HR	1, 11	1.529	0.127	1, 11	1.089	0.371
	F vs. LR	1, 11	0.997	0.446	1, 11	0.439	0.954
	F vs. HR	1, 11	1.365	0.208	1, 11	1.391	0.181
	LR vs. HR	1, 11	0.531	0.903	1, 11	0.727	0.657
36 days	Treatment	3, 11	0.291	0.143	3, 11	1.043	0.401
	C vs. F	1, 11	0.750	0.781	1, 11	1.368	0.178
	C vs. LR	1, 11	1.825	**0.040***	1, 11	0.801	0.685
	C vs. HR	1, 11	1.004	0.424	1, 11	0.618	0.840
	F vs. LR	1, 11	1.908	**0.022***	1, 11	1.478	0.107
	F vs. HR	1, 11	0.883	0.564	1, 11	1.720	**0.043***
	LR vs. HR	1, 11	1.056	0.389	1, 11	0.355	0.988

Supp. Table 3. PERMANOVA table showing differences in the **bacterial** community structure among treatments and specific contrasts of treatments for all sampling dates and at each sampling times separately for riverbed and gravel samples. DF = degree of freedom: numerator, total. F = F value. P = P value.

	**Factors / contrasts**	**Riverbed**			**Gravel Plain**		
		DF	F	P	DF	F	P
All dates	Treatment	3, 83	3.183	**0.001***	3, 83	3.882	**0.001***
	Sampling	6, 83	3.800	**0.001***	6, 83	5.129	**0.001***
	Treatment : sampling	18, 83	0.961	0.576	18, 83	1.269	0.074
4 h	Treatment	3, 11	0.677	0.712	3, 11	0.409	0.997
	C vs. F	1, 11	0.317	0.794	1, 11	0.268	0.860
	C vs. LR	1, 11	0.761	0.537	1, 11	0.609	0.667
	C vs. HR	1, 11	0.847	0.436	1, 11	0.113	0.960
	F vs. LR	1, 11	1.294	0.297	1, 11	0.915	0.510
	F vs. HR	1, 11	0.943	0.461	1, 11	0.122	0.956
	LR vs. HR	1, 11	0.253	0.858	1, 11	0.815	0.578
1 day	Treatment	3, 11	0.383	0.969	3, 11	0.993	0.452
	C vs. F	1, 11	0.863	0.437	1, 11	1.850	0.136
	C vs. LR	1, 11	0.421	0.794	1, 11	0.240	0.895
	C vs. HR	1, 11	0.277	0.902	1, 11	0.231	0.908
	F vs. LR	1, 11	0.338	0.868	1, 11	2.075	0.101
	F vs. HR	1, 11	0.649	0.622	1, 11	1.940	0.132
	LR vs. HR	1, 11	0.109	0.978	1, 11	0.063	0.984
2 days	Treatment	3, 11	0.602	0.755	3, 11	0.621	0.862
	C vs. F	1, 11	0.435	0.680	1, 11	0.549	0.776
	C vs. LR	1, 11	1.577	0.235	1, 11	0.191	0.970
	C vs. HR	1, 11	0.805	0.401	1, 11	0.841	0.509
	F vs. LR	1, 11	0.646	0.503	1, 11	0.302	0.923
	F vs. HR	1, 11	0.239	0.845	1, 11	1.660	0.155
	LR vs. HR	1, 11	0.330	0.802	1, 11	0.613	0.738
8 days	Treatment	3, 11	1.231	0.313	3, 11	2.370	**0.015***
	C vs. F	1, 11	1.005	0.441	1, 11	0.781	0.567
	C vs. LR	1, 11	2.173	0.081	1, 11	5.370	**0.001***
	C vs. HR	1, 11	1.699	0.159	1, 11	2.430	0.046
	F vs. LR	1, 11	0.879	0.493	1, 11	2.393	0.052
	F vs. HR	1, 11	0.150	0.957	1, 11	0.557	0.760
	LR vs. HR	1, 11	1.376	0.290	1, 11	0.844	0.533
12 days	Treatment	3, 11	1.740	0.096	3, 11	4.98	**0.012***
	C vs. F	1, 11	1.483	0.258	1, 11	0.247	0.856
	C vs. LR	1, 11	3.631	**0.018***	1, 11	9.012	**0.001***
	C vs. HR	1, 11	1.723	0.194	1, 11	0.359	0.773
	F vs. LR	1, 11	1.033	0.428	1, 11	5.348	**0.022***
	F vs. HR	1, 11	0.108	0.970	1, 11	0.271	0.844
	LR vs. HR	1, 11	1.668	0.213	1, 11	6.536	**0.005***
28 days	Treatment	3, 11	2.309	**0.044***	3, 11	0.982	0.501
	C vs. F	1, 11	1.445	0.217	1, 11	0.154	0.969
	C vs. LR	1, 11	3.131	**0.027***	1, 11	1.014	0.458
	C vs. HR	1, 11	2.963	**0.035***	1, 11	1.354	0.263
	F vs. LR	1, 11	1.759	0.154	1, 11	0.736	0.631
	F vs. HR	1, 11	0.899	0.473	1, 11	1.272	0.322
	LR vs. HR	1, 11	1.147	0.344	1, 11	1.496	0.215
36 days	Treatment	3, 11	2.247	**0.024***	3, 11	1.254	0.280
	C vs. F	1, 11	1.988	0.074	1, 11	0.076	0.985
	C vs. LR	1, 11	2.910	**0.015***	1, 11	3.197	**0.021***
	C vs. HR	1, 11	3.200	**0.011***	1, 11	0.598	0.705
	F vs. LR	1, 11	0.601	0.729	1, 11	1.802	0.125
	F vs. HR	1, 11	1.260	0.297	1, 11	0.312	0.912
	LR vs. HR	1, 11	1.268	0.299	1, 11	1.772	0.129

Supplementary Figure legend

Supp. Fig. 1. Resilience index Res_t evolution of the Fungal (A and C) and Bacterial (B and D) community structures after the initial disturbance (4h) and over the length of the experiment (1, 2, 8, 12, 28 and 36 days) for the different treatments (F, LR and HR). Points correspond to replicate microcosms for each treatment at each sampling time. Data of each treatment were fitted with robust linear models (RLM).

Supp. Fig. 1

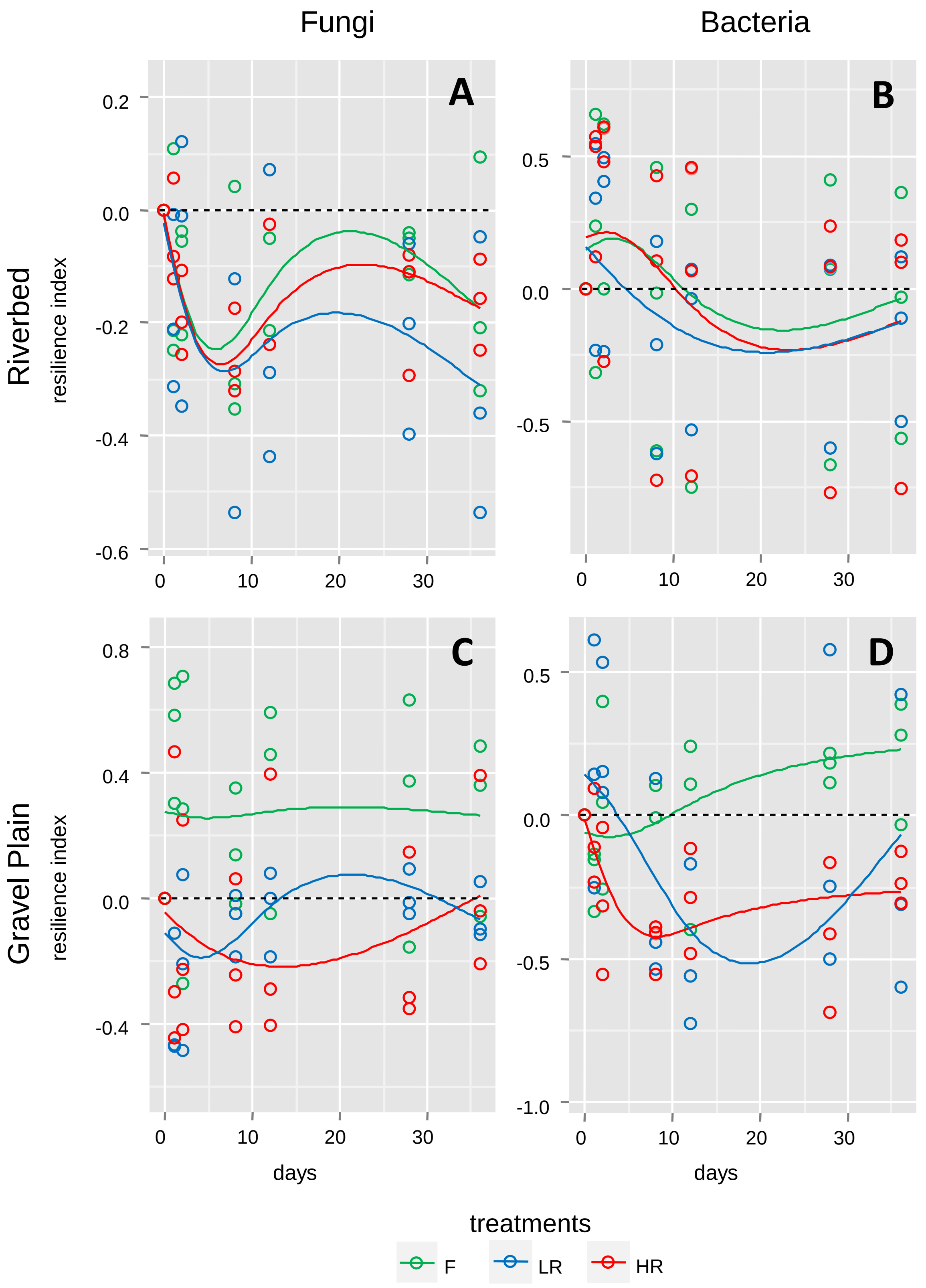

Fungi
Bacteria
A
B
C
D
Riverbed
Gravel Plain
resilience index
resilience index
0.2
0.0
-0.2
-0.4
-0.6
0.5
0.0
-0.5
0.8
0.4
0.0
-0.4
0.5
0.0
-0.5
-1.0
0
10
20
30
days
days
treatments
F
LR
HR
